# A pandemic recap: lessons we have learned

**DOI:** 10.1186/s13017-021-00393-w

**Published:** 2021-09-10

**Authors:** Federico Coccolini, Enrico Cicuttin, Camilla Cremonini, Dario Tartaglia, Bruno Viaggi, Akira Kuriyama, Edoardo Picetti, Chad Ball, Fikri Abu-Zidan, Marco Ceresoli, Bruno Turri, Sumita Jain, Carlo Palombo, Xavier Guirao, Gabriel Rodrigues, Mahir Gachabayov, Fernando Machado, Lostoridis Eftychios, Souha S. Kanj, Isidoro Di Carlo, Salomone Di Saverio, Vladimir Khokha, Andrew Kirkpatrick, Damien Massalou, Francesco Forfori, Francesco Corradi, Samir Delibegovic, Gustavo M. Machain Vega, Massimo Fantoni, Demetrios Demetriades, Garima Kapoor, Yoram Kluger, Shamshul Ansari, Ron Maier, Ari Leppaniemi, Timothy Hardcastle, Andras Vereczkei, Evika Karamagioli, Emmanouil Pikoulis, Mauro Pistello, Boris E. Sakakushev, Pradeep H. Navsaria, Rita Galeiras, Ali I. Yahya, Aleksei V. Osipov, Evgeni Dimitrov, Krstina Doklestić, Michele Pisano, Paolo Malacarne, Paolo Carcoforo, Maria Grazia Sibilla, Igor A. Kryvoruchko, Luigi Bonavina, Jae Il Kim, Vishal G. Shelat, Jacek Czepiel, Emilio Maseda, Sanjay Marwah, Mircea Chirica, Giandomenico Biancofiore, Mauro Podda, Lorenzo Cobianchi, Luca Ansaloni, Paola Fugazzola, Charalampos Seretis, Carlos Augusto Gomez, Fabio Tumietto, Manu Malbrain, Martin Reichert, Goran Augustin, Bruno Amato, Alessandro Puzziello, Andreas Hecker, Angelo Gemignani, Arda Isik, Alessandro Cucchetti, Mirco Nacoti, Doron Kopelman, Cristian Mesina, Wagih Ghannam, Offir Ben-Ishay, Sameer Dhingra, Raul Coimbra, Ernest E. Moore, Yunfeng Cui, Martha A. Quiodettis, Miklosh Bala, Mario Testini, Jose Diaz, Massimo Girardis, Walter L. Biffl, Matthias Hecker, Ibrahima Sall, Ugo Boggi, Gabriele Materazzi, Lorenzo Ghiadoni, Junichi Matsumoto, Wietse P. Zuidema, Rao Ivatury, Mushira A. Enani, Andrey Litvin, Majdi N. Al-Hasan, Zaza Demetrashvili, Oussama Baraket, Carlos A. Ordoñez, Ionut Negoi, Ronald Kiguba, Ziad A. Memish, Mutasim M. Elmangory, Matti Tolonen, Korey Das, Julival Ribeiro, Donal B. O’Connor, Boun Kim Tan, Harry Van Goor, Suman Baral, Belinda De Simone, Davide Corbella, Pietro Brambillasca, Michelangelo Scaglione, Fulvio Basolo, Nicola De’Angelis, Cino Bendinelli, Dieter Weber, Leonardo Pagani, Cinzia Monti, Gianluca Baiocchi, Massimo Chiarugi, Fausto Catena, Massimo Sartelli

**Affiliations:** 1 General, Emergency and Trauma Surgery Department, Pisa University Hospital, Via Paradisa, 2, 56124 Pisa, Italy; 2 Infectious Disease Department, Careggi Hospital, Florence, Italy; 3 Emergency and Critical Care Center, Kurashiki Central Hospital, Kurashiki, Japan; 4 Department of Anesthesia and Intensive Care, Parma University Hospital, Parma, Italy; 5 Hepatobiliary and Pancreatic Surgery Trauma and Acute Care Surgery, Foothills Medical Center, Calgary, AB Canada; 6 Department of Surgery, College of Medicine and Health Sciences, UAE University, Al-Ain, UAE; 7 General Surgery Department, Monza University Hospital, Monza, Italy; 8 General Emergency and Trauma Surgery Department, Bufalini Hospital, Cesena, Italy; 9 Department of Surgery, SMS Medical College and Hospital, Jaipur, India; 10 Cardiology Division 1, Department of Surgical, Medical, Molecular Pathology, and Critical Medicine, School of Medicine, University of Pisa, Pisa, Italy; 11 Department of Surgery, Parc Tauli, Hospital Universitari, Sabadell, Spain; 12 Department of General Surgery, Kasturba Medical College and Hospital, Manipal, Karnataka India; 13 Department of Abdominal Surgery, Vladimir City Emergency Hospital, Vladimir City, Russia; 14 General Surgery Department, Montevideo Hospital, Montevideo, Paraguay; 15 General Surgery, Kavala General Hospital, Kavála, Greece; 16 Antimicrobial Stewardship Program, American University of Beirut Medical Center, Beirut, Lebanon; 17 Department of Surgical Sciences and Advanced Technologies, General Surgery, University of Catania, Cannizzaro Hospital, Catania, Italy; 18 General Surgery, ASUR Marche 5, San Benedetto del Tronto General Hospital, San Benedetto del Tronto, Italy; 19 General Surgery, Mozyr City Hospital, Mazyr, Belarus; 20 General, Acute Care, Abdominal Wall Reconstruction, and Trauma Surgery, Foothills Medical Centre, Calgary, AB Canada; 21 Acute Care Surgery, University Hospital of Nice (CHU de Nice)/Université Côte d’Azur, Nice, France; 22 ICU Department, Pisa University Hospital, Pisa, Italy; 23 General Surgery, University Clinical Center Tuzla, Tuzla, Bosnia and Herzegovina; 24 Servicio de Cirugia General, Universidad Nacional de Asuncion, Hospital de Clinicas Ii Cátedra de Clinica Quirúrgica, Asunción, Paraguay; 25 Dipartimento Di Scienze Di Laboratorio E Infettivologiche, Fondazione Policlinico Universitario Agostino Gemelli Irccs, Roma, Italy; 26 Division of Trauma, Emergency Surgery, and Surgical Critical Care, LAC+USC Medical Center, Los Angeles, USA; 27 Department of Microbiology, Gandhi Medical College, Bhopal, India; 28 General Surgery Department, Rambam Medical Centre, Tel Aviv, Israel; 29 Department of Microbiology, Chitwan Medical College and Teaching Hospital, Bharatpur, Chitwan, Nepal; 30 Harborview Medical Center, University of Washington, Seattle, WA USA; 31 HUS Abdominal Center, Helsinki University Hospital and University of Helsinki, Helsinki, Finland; 32 Trauma and Burns, Inkosi Albert Luthuli Central Hospital and DoH-KZN, Mayville, South Africa; 33 Department of Surgery, Medical School, University of Pécs, Pecs, Hungary; 34 Medical School, National and Kapodistrian University of Athens, (NKUA), Athens, Greece; 35 Department of Translational Research, University of Pisa, Pisa, Italy; 36 Research Institute at Medical University Plovdiv/University Hospital St George, Plovdiv, Bulgaria; 37 Trauma Center, Groote Schuur Hospital, Faculty of Health Sciences, University of Cape Town, Cape Town, 7925 South Africa; 38 Critical Care Unit, Complexo Hospitalario Universitario A Coruña, Instituto de Investigación Biomédica de A Coruña, A Coruña, Spain; 39 General Surgery Department, Zliten Medical Center, Zliten, Libya; 40 Division of Emergency Surgery, Saint-Petersburg Research Institute of Emergency Medicine, Saint-Petersburg, Russian Federation; 41 Department of Surgical Diseases, University Hospital “Prof. Dr. Stoyan Kirkovich”, 2A Gen. Stoletov Str., 6000 Stara Zagora, Bulgaria; 42 Clinic for Emergency Surgery, Belgrade, Serbia; 43 1St General Surgery Unit, Department of Emergency, ASST Papa Giovanni, Bergamo, Italy; 44 Department of Anaesthesia and Critical Care Medicine, Azienda Ospedaliera Universitaria Pisana, Pisa, Italy; 45 General and Emergency Surgery Unit, Sant’Anna University-Hospital, Ferrara, Italy; 46 Department of Surgery No2, Kharkiv National Medical University, Kharkiv, Ukraine; 47 Division of General and Foregut Surgery, University of Milan, IRCCS Policlinico San Donato, Milan, Italy; 48 Department of Surgery, Inje University, Ilsan Paik Hospital, Goyang-si, South Korea; 49 Department of General Surgery, Tan Tock Seng Hospital, Singapore, Singapore; 50 Department of Infectious and Tropical Diseases, Jagiellonian University Medical College, Kraków, Poland; 51 Surgical Critical Care, Department of Anesthesia, Hospital Valdecilla Santander, Santander, Spain; 52 Post-Graduate Institute of Medical Sciences, Rohtak, 124001 India; 53 Department of Digestive Surgery, Centre Hospitalier Universitaire Grenoble Alpes, La Tronche, France; 54 Operative Unit of Anesthesia and Transplant Resuscitation, University of Pisa, Pisa, Italy; 55 Department of Emergency Surgery, Cagliari University Hospital, Cagliari, Italy; 56 Department of Clinical, IRCCS Policlinico San Matteo Foundation, Pavia, Italy; 57 Department of Clinical, Diagnostic, and Pediatric Sciences, University of Pavia, Pavia, Italy; 58 Department of General Surgery, George Eliot Hospital NHS Trust, Warwickshire, UK; 59 Surgery Department, University Hospital, Therezinha De Jesus, Suprema, Brazil; 60 Azienda Ospedaliero Universitaria Di Bologna, Unità Operativa Malattie Infettive, Bologna, Italy; 61 Internal Medicine – Intensive Care, AZ Jan Palfijn Gent, Gent, Belgium; 62 First Department of Anaesthesia and Intensive Therapy, Medical University of Lublin, Lublin, Poland; 63 Department of General, Visceral, Thoracic, Transplant and Pediatric Surgery, University Hospital of Giessen, Giessen, Germany; 64 Department of Surgery, University Hospital Centre Zagreb, Zagreb, Croatia; 65 Department of Public Health, Medical School, University of Naples Federico II, Naples, Italy; 66 Dipartimento Di Medicina, Chirurgia E Odontoiatria, Campus Universitario di Baronissi - Università Di Salerno, Salerno, Italy; 67 Department of General and Thoracic Surgery, University Hospital of Giessen, Marburg, Germany; 68 Department of Surgical, Medical and Molecular Pathology and Critical Care Medicine, University of Pisa, Pisa, Italy; 69 General Surgery Department, Istanbul Medeniyet University, Istanbul, Turkey; 70 Department of Medical and Surgical Sciences – DIMEC, Alma Mater Studiorum - University of Bologna, Bologna, Italy; 71 General Surgery of the Morgagni - Pierantoni Hospital, Forlì, Italy; 72 Pediatric Intensive Care Unit, Department of Anesthesia and Intensive Care, ASST Papa Giovanni XXIII, Bergamo, Italy; 73 Hepato-Billiary-Pancreatic (HPB) Surgery Center, Emek Medical Center, Afula, Israel; 74 Emergency County Hospital of Craiova, Craiova, Romania; 75 Mansoura Faculty of Medicine, Mansoura University, Mansoura, Egypt; 76 The Ruth and Bruce Rappaport Faculty of Medicine, Technion – Israel Institute of Technology, Rambam Health Care Campus, Haifa, Israel; 77 Department of Pharmacy Practice, National Institute of Pharmaceutical Education and Research (NIPER), Hajipur Vaishali, Bihar, India; 78 Department of Surgery, Riverside University Health System, Moreno Valley, CA USA; 79 School of Medicine, Loma Linda University, Loma Linda, CA USA; 80 Shock Trauma Center at Denver Health, Denver, CO USA; 81 Department of Surgery, Tianjin Nankai Hospital, Nankai Clinical School of Medicine, Tianjin Medical University, Tianjin, China; 82 Hospital Santo Tomás, Panama City, Panama; 83 Trauma and Acute Care Surgery Unit, Hadassah - Hebrew University Medical Center, Jerusalem, Israel; 84 Department of Biomedical Sciences and Human Oncology, University of Bari, Bari, Italy; 85 University of Maryland School of Medicine, Baltimore, MD USA; 86 Intensive Care Unit, University Hospital of Modena, Modena, Italy; 87 Scripps Clinic Medical Group, La Jolla, CA USA; 88 Department of Respiratory and Critical Care Medicine, University Hospital Giessen, Giessen, Germany; 89 Department of General Surgery, Military Teaching Hospital, Hôpital Principal de Dakar, Dakar, Senegal; 90 Division of General and Transplant Surgery, University of Pisa, Pisa, Italy; 91 Emergency Medical Department, Pisa University Hospital, Pisa, Italy; 92 Department of Emergency and Critical Care Medicine, Saint-Marianna University School of Medicine, Kawasaki, Japan; 93 Department of Surgery, Amsterdam University Medical Center, Amsterdam, The Netherlands; 94 Professor Emeritus, Virginia Commonwealth University, Richmond, VA USA; 95 Infectious Diseases Section, King Fahad Medical City, Riyadh, Kingdom of Saudi Arabia; 96 Immanuel Kant Baltic Federal University, Regional Clinic Hospital, Kaliningrad, Russia; 97 Department of Internal Medicine, University of South Carolina School of Medicine, Prisma Health-Midlands, Columbia, SC USA; 98 Department of Surgery, Tbilisi State Medical University, Kipshidze Central University Hospital, Tbilisi, Georgia; 99 Department of Surgery, Bizerte Hospital, Bizerte, Tunisia; 100 Faculty of Medicine, Tunis University, Tunis ElManar, Tunisia; 101 Division of Trauma and Acute Care Surgery, Department of Surgery, Universidad del Valle, Cali, Colombia; 102 General Surgery Department, Emergency Hospital of Bucharest, Carol Davila University of Medicine and Pharmacy, Bucharest, Romania; 103 Department of Pharmacology and Therapeutics, College of Health Sciences, Makerere University, Kampala, Uganda; 104 King Saud Medical City, Ministry of Health and College of Medicine, AlFaisal University, Riyadh, Kingdom of Saudi Arabia; 105 National Public Health Laboratory, Khartoum, Sudan; 106 General Surgery, University of Health Sciences, Adana City Training and Research Hospital, Adana, Turkey; 107 Infection Control Coordinator, Hospital de Base Do Distrito Federal /IGESDF, Brasilia, Brazil; 108 Department of Surgery, Trinity College Dublin, Dublin, Ireland; 109 Infection Prevention and Control Unit, Centre des Massues, French-Red Cross, Lyon, France; 110 Department of Surgery, Radboud University Medical Center, Nijmegen, The Netherlands; 111 General Surgery, Dirghayu Pokhara Hospital, Pokhara, Nepal; 112 Department of Emergency, Digestive and Metabolic Minimally Invasive Surgery, Centre Hospitalier Intercommunal de Poissy, Saint Germain en Laye, France; 113 ICU Department, Bergamo Hospital, Bergamo, Italy; 114 Orthopedic and Traumatology Department, Pisa University Hospital, Pisa, Italy; 115 General Surgery, Department, Henri Mondor Hospital, Paris, France; 116 Department of Surgery, John Hunter Hospital, Newcastle, Australia; 117 University of Newcastle, Callaghan, Australia; 118 General Surgery, Royal Perth Hospital, The University of Western Australia, Perth, Australia; 119 Infectious Diseases Unit, Bolzano Central Hospital, Bolzano, Italy; 120 Radiology Department, Gavazzeni Hospital, Bergamo, Italy; 121 General Surgery Department, Cremona Hospital, Cremona, Italy; 122 General Surgery Department, Macerata Hospital, Macerata, Italy

**Keywords:** Pandemia, International, Thoughts, Reflection, Ethics, Biology, Politics, Health care, Policy

## Abstract

On January 2020, the WHO Director General declared that the outbreak constitutes a Public Health Emergency of International Concern. The world has faced a worldwide spread crisis and is still dealing with it. The present paper represents a white paper concerning the tough lessons we have learned from the COVID-19 pandemic. Thus, an international and heterogenous multidisciplinary panel of very differentiated people would like to share global experiences and lessons with all interested and especially those responsible for future healthcare decision making. With the present paper, international and heterogenous multidisciplinary panel of very differentiated people would like to share global experiences and lessons with all interested and especially those responsible for future healthcare decision making.

The term Public Health Emergency of International Concern is defined by the World Health Organization (WHO) as “an extraordinary event which is determined, as provided in these regulations: to constitute a public health risk to other states through the international spread of disease; and to potentially require a coordinated international response.” This definition implies a situation that is serious, unusual and/or unexpected. Furthermore, it may carry implications for public health beyond the affected state’s national border and may require immediate international action.

On January 30, 2020, following the recommendations of the Emergency Committee, the WHO Director General declared that the outbreak constitutes a Public Health Emergency of International Concern.

From that date, unprecedented events transpired, and the world’s population experienced a multitude effect (physical, psychological, sociological and often economic) from this new and aggressive virus, the Sars-Cov-2.

The world has faced this crisis and continues to deal with it.

As said “Successfully overcoming a crisis does not only mean successfully eliminating its consequences and reducing its negative impact. Overcoming a crisis also involves realizing one’s own mistakes, and shortcomings to respond to the ongoing crisis and constantly work on combating it. We will truly overcome the crisis if we take something from it and if we learn lessons that will allow us to be more prepared and resilient when we have to face a new risk in the future.”

Profound modification in the perception of a life and its value has occurred in the general population, together with the change in perception of the condition considered well-being. The WHO definition of health as complete well-being no longer applies for this for purpose.

In our opinion, some critical points must be stressed to inform present and future generations to discuss and plan.

The present paper represents a white paper concerning the tough lessons we have learned from the COVID-19 pandemic. Thus, an international and heterogenous multidisciplinary panel of very differentiated people would like to share global experiences and lessons with all interested and especially those responsible for future healthcare decision making.

## What has been learnt from ongoing pandemic?

### 1st

#### To build resilient health systems

The most important element of pandemic preparedness is a resilient health system to rapidly detect, assess, report, and respond to novel outbreaks. Although most high-income countries have robust health systems, they often lacked sufficient capacity to treat large numbers of patients with COVID-19 or protect health workers from the infection at the beginning of the pandemic. Moreover, in the pandemic situation, they must share their knowledge and tools with those who unfortunately are less equipped, with a win–win vision.

We experienced many challenges regarding the lack of several fundamental pieces of organizational structure [1]. In subsequent phases, moreover, health systems also demonstrated the impossibility to give people the access to the necessary treatment and screening strategies because of the exhaustion of resources in facing the initial crisis. In fact, no organizational models were prepared to relocate underutilized forces to their duties, thus reducing worldwide the power of programs for prevention and cure, such as those for neoplastic and chronic diseases such as cardiovascular, pulmonary and neurologic. This resulted in a reduction of assistance of these patients. Lower-income regions experienced complete collapse of their health and economic systems.

### 2nd

#### To invest in vaccination diffusion and protocols

Prevention is the most cost-effective way to maintain the health of the population in a sustainable manner. The ongoing COVID-19 pandemic is a reminder of the importance of vaccination. We already know that vaccination is one of the most impactful and cost-effective public-health interventions. It has eradicated smallpox, nearly eradicated polio and, in recent decades, reduced the incidence of infectious diseases that once killed millions every year, such as measles, helping to halve child mortality. It is concerning though that it has been stated that if similar practices involving scientific misinformation and social media had existed in previous generations, these diseases would never have been successfully addressed. It is now accepted that the only way to end the COVID-19 pandemic, minimize loss of life and return to some semblance of normality is through vaccination. Maximizing the vaccination diffusion and vaccine accessibility is of upmost importance whatever the cost.

The diffusion of the vaccination programs, in many developed areas, has been vigorously opposed by the activities of antivaccination movements (“Antivax activists”). A vaccine refusal attitude has spread mainly where high-resource settings exist, and—de facto—a lower incidence of lethal infectious diseases is present. As a paradoxical counterpart, in low-resource settings the vital importance of vaccination is typically well accepted. The White Paper authors believe it is fundamental to adhere to unequivocal communication about the benefits of broad vaccination campaigns and guarantee the most transparent and comprehensible information about funding, production, testing and study results. Social media and traditional media should cooperate in reducing the spread of sensationalistic and fake news. Further, we suggest that social scientists should be urgently recruited and embraced by physicians to help understand and investigate how scientific misinformation can be so pervasive in modern society. Politicians and governments should also be held accountable to make courageous decisions to fulfill their mandates to serve and protect their constituents, rather than seeking votes through supporting and even promoting popular but unfounded theories.

### 3rd

#### To defend the integrity of science

The pandemic has provided to medical science in general the first true battlefield to test itself since the start of the century. Science won some important battles against the Sars-Cov-2 but seems to have lost others against unfounded social media campaigns.

Unequivocally, the greatness of the scientific progress and the presence of great researchers enabled societies to understand the virus, its modes of transmission, and most effective public health interventions. Sharing the information, enhanced by the network, allowed immediate confrontation among different experiences and professionals. After an immediate viral sequencing, safe and effective vaccines have been developed and approved at record speed, giving us a crucial new way to protect people from the virus, in addition to traditional public health measures.

Unfortunately, the medical community was immediately required to satisfy the tsunami of questions arising from the media. For the first time in ages, medical progress has been on the front pages of every newspaper, for more than one year.

Due to the peculiar approach to information we nowadays have, the scientists tried to give precise and comprehensible instructions in any available moment, with transparency and clarity, basing on precedent works or, in lack of these, on common sense. But we all forget that the time of science and research is not the time of media and news. Instead of developing a new public conscience about how careful and high class the research work is and should be, we tried to pair up with the media and social media timetable, exposing the scientific production to bias and liability.

Mass information, moreover, has necessities and characteristics that are very dangerous for science: It should be quick, obtain an emotional response and, sometimes, apply to a precise, politically oriented vision of the reality.

Through sometimes manipulating their good faith and others through “legitimizing” unqualified physicians, “experts” were selected and used as political flags to represent different ideas about the management of restrictions and openings, and not based on their actual expertise. The oversimplification of concepts, necessary to give general indications, led to the creation of slogans and factions, taking away energies and time for an appropriate in-depth analysis. Some ideas have been empowered because they were answering to media needs and not to scientific real necessity.

The spontaneous response of the academic community scientific production in the first phase of the pandemic was remarkable. However, the vast quantity of articles proposed, the willingness to acquire a scientific leading role, and the emergent need of research stressed the efficacy of the peer review process, leading to the regrettable retraction of some papers, even from top level journals.

The lack of coordination and ethics endangered the circulation of adequate and true scientific information, both on the media and on the research side. Moreover, the will to appear sometimes buried down medical ethics and professional behavior codes. For the rapidity of answers and collaboration to contribute in solving the disaster, we will definitely remember this period as representative and extraordinary. However, it will be also remembered as a black period of medical sciences, when some figures tried to get personal gain from general crisis.

Lastly, the impossibility of an international coordination has been exacerbated by the mix of commercial interests, public funds and organizational issues linked to vaccine production, experimentation and diffusion. The crisis also highlighted the need for supranational Institutions, (i.e., EU), to take back a direct control of strategic activities such as that of drug development and production. Moreover, the public financing system should insist from the industries the necessity to adhere to strict protocols not allowing for unfair price changings and economical extortion.

### 4th

#### To abolish rhetoric and adhere to truth

Truth and medical authority usually come together, but which one is the source of the other?

Since the start of the past century, people’s expectation on medicine and faith in science have grown to an unseen level, this being a direct result of the improving outcomes. But in the last decades objections and passionate reactions against unlimited progress and its cost also started to appear. In the same way, patients have begun to question what is proposed them by the medical community, becoming more and more aware about their health and their choices. In the same way, the mixture of science and pseudoscience began to be part of the everyday debate. Patients have developed an opinion on the medical reliability, based on the personal experiences, the access to information and the ability to discriminate it. The trustworthiness and the clarity of the medical system, along with its ability to communicate, are of course directly involved in this process, both in positive and negative manner.

Considering the diffusion of the pandemic and its burden, almost everyone in the world played the role of the potential patient, thus leading to an increased request of proper information.

In starting a trustworthy dialogue and counseling with a person, it is fundamental to adhere to intellectual honesty, clearness and empathy, avoiding paternalism, rhetoric and sensationalism. The rules are not different when it comes to answer the questions of an entire population.

Furthermore, the chance to increase the recipients of information gives to the communicator an additional responsibility: It is fundamental to educate on how to read, understand and be prepared. Ideally, doctors should be able to give adequate and personalized instruments to patients. In fact, we must not "playing with the people as [with] children, just trying to satisfy them careless if they become better or worse" [2]. We must proceed to give patients the ability not only to interpret what is going to happen, but also to recognize the complexity and the ramification of the path leading to the outcome. In the same way, in times such as these, we should clearly instruct people on what we can achieve, what we know at present and what it is—for now—outside of our range of control and comprehension. Only in this way, as a cohesive community, we can start a new era of cooperation and trust. Otherwise, the response will be always inconsistent and vague, with consequent misunderstanding, disappointment and confusion.

### 5th

#### To focus on equity

As United Nations High Commissioner for Human Rights Michelle Bachelet commented “Human rights need to be front and center in response (…) to effectively combat the outbreak means ensuring everyone has access to treatment, and is not denied health care because they cannot pay for it or because of stigma (…)” [3].

Even before the coronavirus pandemic, social, economic and health inequities were the prevailing global narrative. COVID-19 amplified long-standing systemic inequalities, including access to health care. Infectious diseases should affect populations similarly, but COVID-19 demonstrated that low-income people, those who are less educated, and ethnical minorities are disproportionately affected. Of course, inequality is not a mere geographical concept. Within the same nations, and even inside the same cities, deep disparities exist between different groups. As known, health care is strictly linked to the economic and cultural status of people and countries.

Singer created the term “sindemic” to underline the amplifying effect of the relationship between the socio-sanitary status and the impact of an infectious disease. As highlighted by Horton [4], sindemics “are characterized by biological and social interactions between conditions and states, interactions that increase a person’s susceptibility to harm or worsen their health condition.” In other words, not even in our own country can we consider ourselves as a homogeneous population: The differences among socioeconomic classes make it impossible to predict a unique response to the pandemic and create dramatically different clusters of morbidity-mortality. All of these are risk factors for an uncontrolled development of a pandemic and for a deepening of the inequities. Even where disparities will continue to exist the continuation of present status is risky and almost unacceptable. International organizations must prepare an action plan to moderate disparities and increase the access to fundamental health support for as many people as possible, starting from prevention. Whenever single governments resist the necessary actions to limit the disease spread, they must be forced to proceed on this path. Most exposed and weak peoples must not pay in person for inequalities and disproportion in culture and health care, remembering that a healthy population in its entirety is a safer population. Funding solutions that reduce the risk of patient financial destitution are essential in accomplishing this.

### 6th

#### To look at health care from a global perspective

Since the rise of globalization, the world has become more closely connected and people can easily interact without facing barriers. The free movement of people, goods, and services brought about by globalization has stimulated socioeconomic development, but it has also become a channel for diseases spreading. We cannot continue to consider the world as a set of separated and not mutually influenced nations. It is a matter of facts that we all live on the same planet, and we are responsible for one other without exceptions. No borders exist for diseases and no borders must exist for basic and fundamental health care. The necessity to overcome the actual distribution of health care assistance is becoming day by day more impellent.

Moreover, as already written, sindemia and sociocultural differences lead to different diffusion and effects of infectious rapid spreading infectious disease [4]. Some classes of population in many countries have not the same possibilities to access to the different healthcare facilities. Consequently, they are more exposed to the effect of chronic illness and to the rapid spreading and lethal effects of new onset infectious diseases. This not only impacts them but directly also affects the other population clusters who may have access to health care facilities but are as well exposed to the risk of infection. This non-inclusive organizational model has shown its fragility and ineffectiveness even in some of the so-called top 10 healthcare systems of the world.

The difficulties shown by the international organization in facing the first steps of pandemic have been evident. WHO was not able to keep up with the events, but its potential to act was even heavily influenced by politico-economical events.

The panic reaction of the first moments of pandemic showed the weakness of the organizational model. During ordinary situations, in fact, the cooperation seemed to work, but during this dramatic situation witnessed the cancellation of financial assistance to WHO from some Nations brought out all the hidden consequences. Even in a situation during which the international agencies have shown lack of preparedness and ineffective action, their overcoming and partial or total de-authorization by single nations is not acceptable.

Moreover, unequal distribution of vaccine doses throughout the world happened even within the higher income countries. Considerations about the cause: if this was the consequence or the political trigger for changing the WHO support policies is out of the aim of present paper. For sure in a necessary global perspective, the actual trend in promoting universal diffusion in vaccination distribution appears antithetic to the previous international behaviors.

Subjecting large numbers of people in certain low-income countries to economic sanctions that limit imports of vaccines, life-saving medications, and other medical equipment due to political conflicts or disagreements is immoral and unethical. It violates the basic human rights advocating that all humans are equal. Healthcare professionals and all respected medical societies and organizations should stand firmly against these sanctions that put innocent people’s lives at risk and imply that their lives are less important than others.

Last but not least, sharing of health systems tools and facilities sharing must not be promoted in a subtle new form of colonization. In fact, healthcare facilities, structures and tools distribution from high-income systems to lower ones must be promoted together adequate and proportional growth in infrastructure. Healthcare promotion cannot be done without infrastructural, social and territorial implementation to allow the heath care actions to be effective.

### 7th

#### To support “One globe one health” approach

To manage the ongoing pandemic crisis and to better anticipate the next one, it is of outmost importance to strengthen the foundations of an ecology of health, focusing on the interdependencies between the functioning of ecosystems, sociocultural practices and the health of human, animal and plant populations taken together. The system within we live in is a closed system. The more we dysregulate it, the more violent will be the reset to equilibrium.

So, the true prevention starts from understanding the deep connection among environment, use of resources and uncontrolled development. Even considering only the economical perspective and not the moral one, the cost of facing future subsequent pandemics could be even higher than reconsider worldwide the paradigm of an unstoppable economic growth.

Even considering the economical perspective and not the moral one, the cost of facing future subsequent global pandemics could be higher than the planned and anticipated economical gain that do not consider this approach.

### 8th

#### To make digital health technologies a channel for delivering primary health care

The COVID-19 pandemic is transforming the global health community’s acceptance and use of digital health technologies. The need to monitor the data on number of patients with related diagnoses before the first case of a patient with this type of disease is identified. If there are anomalies, then specific actions should be taken to identify the cause of this variation. For this reason, technological infrastructure must be fairly organized and shared within nations worldwide, with respect for individual rights.

COVID-19 has forced many countries to social distancing to prevent the spread of the epidemic. Building more robust telemedicine systems in hospitals and communities worldwide is becoming very important. This will reduce at first the pressure on the hospital infrastructure and resources such as beds and healthcare workers. Secondly, those who are healthy may stay at home without going to the doctor’s office unnecessarily but receiving assistance as well if needed. Thirdly, people who are ill but can be treated online by qualified physicians without the need for special medical equipment may receive prompt and cost-effective care and counseling.

### 9th

#### To protect healthcare workers

The most outrageous failure of many health systems has been, and continues to be, the inability to adequately protect professionals and healthcare workers. Many thousands of healthcare workers have been infected and died amid the ongoing coronavirus outbreak, a sign of the immensely difficult working conditions for doctors, nurses, prehospital providers and healthcare workers in general. They should instead be among those best protected. Moreover, ultra-specialized branches of a health system providing a unique service that cannot be performed by other medical disciplines must be secured in a protected environment, minimizing their risks of acquiring the disease. In fact, the consequent eventual lack of experienced teams cannot be faced simply by reintegrating retirees or replenishing the ranks with new levers.

Recognizing teamwork under stress and possible burnout, promoting effective communication and conflict resolutions, recognizing and training leadership, mental health support were key behavioral and psychological elements that were proven to play an important role in supporting the performance, health and safety of healthcare teams [5].

The importance of effective team dynamics and nontechnical skills training and predefined processes and protocols should be highlighted and implemented for facing future crisis.

### 10th

#### To be prepared for mass casualties during pandemic

The COVID-19 pandemic has continued to request resources implementation for nearly two years. At present and in the last months, the different systems have not shown an adequate capability to face an eventual sudden increase in request as in a mass casualty scenario. Moreover, millions of diagnoses are missed from the count, meaning that the health system has not been able to guarantee the standard of care we were all used to. The effect of the pandemic on population exceeds the simple count of infected, hospitalized and dead patients: Every day of difficulty in guaranteeing the healthcare standard brought direct consequences on the non-COVID patients.

Trauma and acute care services have been involved in the preparedness, planning and response to the COVID-19 pandemic. During the COVID-19 pandemic, hospital leadership and health providers have faced difficult decisions in critical resource provision and the triage of patients in need of intensive care beds, mechanical ventilation and extracorporeal membrane oxygenation (ECMO). Supplying PPE and isolating patients and affected staff have been essential to infection control and have tested hospital resources. Decontamination, isolation, triage and the rapid mobilization of health personnel to provide expert care is exactly what trauma and emergency surgeons train for and undertake in mass casualty incidents. Rapid triage and patient flow during mass casualty incidents via a unidirectional dedicated in-hospital route toward specialist care closely resemble the route used for patients with suspected or confirmed COVID-19: from the triage area to a designated ED zone and on to a definitive treatment area. All staff who come into contact with a COVID-19 patient must perform a strict chain-of-command decontamination process with attention to hand hygiene and PPE. Thus, medical personnel who had MCI training are likely to pay dividends in preparedness for disaster, whether a pandemic, natural disaster or conflict.

The first step to avoid the complete debacle of the complex health system organization is to understand which basic level of care is not negotiable, along with the resources needed to maintain it. Even facing a crisis like a pandemic, there must be always a chance to respond to a mass casualty incident, and this can be allowed only by thinking the healthcare facilities and healthcare professionals as a modular system. The rapid dislocation of modular resources, in order to spare and guarantee the functioning of the basic level of assistance, is fundamental to reach the flexibility needed to be prepared for an unexpected increased request, as in mass casualty situations. To this purpose, it is mandatory to shift the decisional chain from a unidirectional one with bureaucrats providing guidelines to professionals, to a paritetic and interactive one, where administrators/stakeholders and health professionals are used to reciprocal interactions and exchange of information, leading to responsible and accomplished decisions [6].

The investigation of the truth is in one way hard, in another easy. An indication of this is found in the fact that no one is able to attain the truth adequately, while, on the other hand, no one fails entirely, but everyone says something true about the nature of things, and while individually they contribute little or nothing to the truth, by the union of all a considerable amount is amassed. Therefore, since the truth seems to be like the proverbial door, which no one can fail to hit, in this way it is easy, but the fact that we can have a whole truth and not the particular part we aim at shows the difficulty of it. Perhaps, as difficulties are of two kinds, the cause of the present difficulty is not in the facts but in us.

Aristotele (384–322 BC), *Metaphysic.*

## Data Availability

Not applicable.

## Abbreviation

WHO: World health organization
